# The cytomegalovirus protein UL138 induces apoptosis of gastric cancer cells by binding to heat shock protein 70

**DOI:** 10.18632/oncotarget.6800

**Published:** 2015-12-30

**Authors:** Wenjing Chen, Kezhi Lin, Liang Zhang, Gangqiang Guo, Xiangwei Sun, Jing Chen, Lulu Ye, Sisi Ye, Chenchen Mao, Jianfeng Xu, Lifang Zhang, Lubin Jiang, Xian Shen, Xiangyang Xue

**Affiliations:** ^1^ Department of General Surgery, First Affiliated Hospital, Wenzhou Medical University, Wenzhou, China; ^2^ Experimental Center, Wenzhou Medical University, Wenzhou, China; ^3^ Department of Microbiology and Immunology, Institute of Molecular Virology and Immunology, Institute of Tropical Medicine, Wenzhou Medical University, Wenzhou, China; ^4^ Department of Rheumatology, First Affiliated Hospital, Wenzhou Medical University, Wenzhou, China; ^5^ Key Laboratory of Molecular Virology and Immunology, Unit of Pathogen-Host Interaction and Epigenetics, Institut Pasteur of Shanghai, Shanghai, China

**Keywords:** gastric cancer, cytomegalovirus, UL138, apoptosis, HSP70

## Abstract

It has been hypothesized that human cytomegalovirus (HCMV) could act as a tumor promoter and play an “oncomodulatory” role in the neoplastic process of several human malignancies. However, we demonstrate for the first time that UL138, a HCMV latency-associated gene, could act as a tumor inhibitor in gastric cancer (GC). The expression of UL138 is down-regulated in HCMV positive gastric adenocarcinoma tissues, especially in poorly or none differentiated tumors. Overexpression of UL138 in several human GC cell lines inhibits cell viability and induces apoptosis, in association with the reduction of an anti-apoptotic Bcl-2 protein and the induction of cleaved caspase-3 and caspase-9. Moreover, protein array analysis reveals that UL138 interacts with a chaperone protein, heat shock protein 70 (HSP70). This interaction is confirmed by immunoprecipitation and immunostaining *in situ* in GC cell lines. In addition, this UL138-mediated cancer cell death could efficiently lead to suppression of human tumor growth in a xenograft animal model of GC. In conclusion, these results uncover a previously unknown role of the cytomegalovirus protein UL138 in inducing GC cells apoptosis, which might imply a general mechanism that viral proteins inhibit cancer growth in interactions with both chaperones and apoptosis-related proteins. Our findings might provide a potential target for new therapeutic strategies of GC treatment.

## INTRODUCTION

Human cytomegalovirus (HCMV) is a multifaceted beta-herpesvirus that leads to a life-long persistence in a highly host-specific manner. Its infection frequency ranges between 50%–100% in adult population [1]. HCMV causes severe and fatal diseases in immunocompromised individuals including AIDS patients and recipients of organ transplants. During the last decade, increasing evidences indicated that HCMV was associated with several human malignancies, including colorectal carcinoma, malignant glioma, skin cancer and prostate cancer [2–8]. Detection of viral mRNA, DNA, or antigens in tumor tissues suggested a role of HCMV infection in etiology of human malignancies. HCMV has been hypothesized for decades to play an “oncomodulatory” role in the neoplastic process, considering that HCMV infection could induce cellular responses stimulating growth of neoplastic cells [9, 10]. Consistently, HCMV suppress host translation machinery [11], and HCMV gene products have also been demonstrated to dysregulate cell cycle progression, block apoptotic pathways, cause DNA damages and inhibit tumor suppressive proteins [9, 12–14]. However, the clinical relevance of these experimental findings remains controversial, including low levels of HCMV nucleic acids, lack of HCMV mRNA or the absence of viral replication observed in tumors [15–17]. Moreover, it has recently been shown that acute leukemia cells infected by HCMV could inhibit their proliferation and induced apoptosis, although HCMV induced significant up-regulation of an anti-apoptotic gene cFLIP and down-regulation of pro-apoptotic genes p53 and Gadd45gamma [18].

HCMV encodes 252 open reading frames in 230-kb genome and is believed to encode approximately 180 proteins. Herpesvirus switches its acute infection state to latent infection when facing competent immune system of hosts. During the latent stage, the viral genome persists in the infected cells with limited viral proteins including LUNA, UL133-UL138 locus, US28, UL111A and the cytomegalovirus latency-associated transcripts (CLTs), whereas no detectable virus production [19, 20]. Among these latency-associated genes, the UL133–UL138 locus in the ULb' region encodes four viral proteins: UL133, UL135, UL136 and UL138, which are essential for viral replication and exist in clinical isolates [21], in association with immune evasion, and virus dissemination in host cells [22], especially UL136 is expressed as five protein isoforms with unique roles in replication [23]. It has also been found that UL133-UL138 genes mediated context-dependent outcomes of infection [22]. Our previous study has demonstrated that HCMV infection exists in gastric cancer and its latent infection is associated with the development of gastric cancer [24]. In addition, we found that the UL138 is widely expressed in almost all tumor tissues. However, pathological roles of this viral protein in cancer cell growth remains largely unknown. It has been reported that the UL138 could enhance the expression of tumor necrosis factor receptor 1 (TNFR1) [25, 26] on the cell surface, but decrease the expression of multidrug resistance-associated protein-1 (MRP1) [27]. Therefore we hypothesized that UL138 might contribute to the control of gastric cancer development.

In the present study, we identified that UL138 was down-regulated in human gastric adenocarcinoma tissues, especially in poorly or none differentiated tumors. In addition, overexpression of UL138 greatly induced apoptotic cell death in different gastric cancer cells, in association with increased cleavage of apoptotic proteins caspase-3 and caspase-9 and reduction of an anti-apoptotic protein Bcl-2. We further identified that UL138 could form a complex with HSP70 and several other cancer-related proteins to inhibit development of gastric cancer. More importantly, we confirmed that UL138 could significantly suppress tumor growth *in vivo* in a xenograft animal model of gastric cancer. Our findings reveal a critical role of the HCMV protein UL138 in cancer cell death.

## RESULTS

### Down-regulation of UL138 expression in human gastric adenocarcinoma

**Figure 1:**
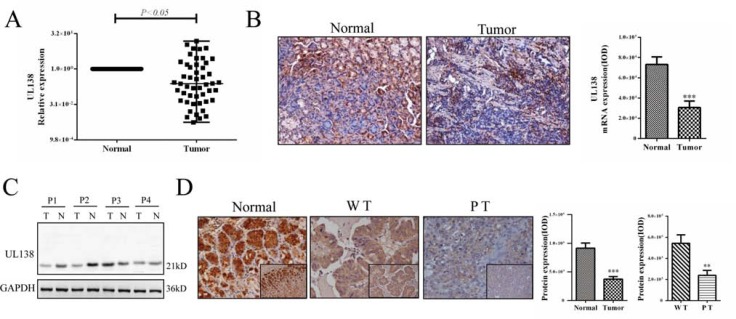
Expression of UL138 is down-regulated in human gastric cancer tissues (**A**) UL138 transcripts were measured by quantitative real-time PCR in the tumor and paired adjacent non-neoplastic (Normal) specimens of 49 HCMV positive gastric cancer samples. The relative expression of UL138 was normalized to GAPDH (2^−ΔΔCt^). *P value* was obtained by using a paired Student's *t*-test. *P < 0.05. Median expression level in each sample group was indicated by a horizontal line. The melt curve was in Figure S2B. (**B**) Expression of pUL138 transcript in gastric tissues was confirmed by *in situ* hybridization technique under microscope (magnification, × 200). The specificity of the probe was showed in Figure S3. The right picture showed ISH result measured by Image-Pro Plus software. *n =* 49. Different UL138 mRNA expression in tumor (Tumor) and adjacent non-neoplastic (Normal) tissues was showed in the integral optical density (IOD). ***P < 0.001. (**C**) The levels of UL138 protein expression in 4 pair samples were detected using anti-UL138 specific antibody by Western blotting. P1–P4 indicated the different patients with gastric cancer. T, tumor; N, adjacent normal tissue. (**D**) Expression and localization of pUL138 in variously differentiated gastric cancer tissues were performed by immunohistochemistry (magnification, × 200 and × 400). Adjacent normal tissue was used as control (magnification, × 200 and × 400). The specificity of the IHC detection was showed in Figure S4. UL138 protein in different tissues (*n =* 49) were measured by semi-quantitative immunohistochemistry. WT, well differentiated tumors; PT, poorly or none differentiated tumors. **P < 0.01, ***P < 0.001.

Our previous study has demonstrated that UL138 broadly expressed in the tissues of gastric cancer and corresponding normal tissues [24]. To investigate the potential effects of UL138 during development of human gastric cancer, quantitative real-time PCR, *in situ* hybridization (ISH), Western blotting (WB) and immunohistochemical (IHC) techniques were utilized to determine the expression level of UL138 in 49 human gastric cancer tissues and corresponding adjacent normal tissues (Figure S1). As shown in Figure 1A, the UL138 transcript in tumor samples was significantly lower than those in adjacent normal tissues (*P < 0.05*), consistent with the ISH observation (Figure 1B). Compared with normal tissues, tumors had lower level of UL138 not only in RNA transcription but also in protein expression (Figure 1C and 1D). We next explored the relationship between UL138 expression and clinical pathological characteristics of gastric cancer (Table 1). Clinical data showed that the expression level of UL138 was associated with the tumor differentiation (*P = 0.025*) in gastric cancer patients. As shown in Figure 1D, there was a significant increase of UL138 expression in well or moderately differentiated tumors (WT), compared with poorly or none differented tumors (PT). However, no relationship was obviously observed among UL138 expression and gender, age, tumor size, tumor location, macroscopic type or TNM GC classification (Table 1).

**Table 1:** Clinical-pathologic features and UL138 expression of 49 specimens

Clinicopathologic variables		*n*	UL138^C^	*P* value^d^
Gender	Male	43	7.56 (0.715∼38.87)	0.625
	Female	6	13.60 (4.42∼35.83)	
Age	< 60 years	13	18.42 (3.18∼41.03)	0.483
	≥ 60 years	36	6.60 (0.75∼36.23)	
Tumor location	Upper third	7	5.53 (0.066∼72.45)	0.774
	Middle third	12	9.58 (1.69∼26.71)	
	Lower third	27	7.56 (0.85∼40.70)	
Tumor size	< 5 cm	24	12.16 (1.04∼37.61)	0.650
	≥ 5 cm	24	5.81 (0.78∼27.73)	
Differentiation	Poorly or none	35	6.09 (0.58∼19.39)	**0.025**
	Well or moderately	12	48.72 (4.84∼111.07)	
Primary tumor^a^	T1/2	11	7.56 (0.577∼19.40)	0.487
	T3/4	38	10.74 (0.95∼39.32)	
Regional lymph nodes^a^	N0	16	8.27 (0.65∼35.99)	0.183
	N1	9	28.33 (6.45∼55.77)	
	N2	12	4.1 (0.18∼15.48)	
	N3	11	17.01 (4.74∼87.16)	
Anatomic stage^a^	Stage I	9	7.12 (0.57∼38.99)	0.388
	Stage II	12	11.88 (2.49∼35.99)	
	Stage III	25	8.16 (0.85∼28.71)	
	Stage IV	2	63.01 (38.87∼87.16)	
CA19–9^b^ (U/ml)	≤ 37	34	11.88 (0.58∼60.81)	0.97
	> 37	6	15.87 (6.14∼24.80)	

a according to NCCN Guideline Version 2.2012 Gastric Cancer Staging.

b carbohydrate antigen 19–9.

c UL138 expression was shown as Protein IOD (integrated optical density) × 10^−4^, median of relative expression with 25th–75th percentile is listed in parentheses.

d *P value* < 0.05 is in bold.

### Overexpression of pUL138 induces apoptosis in gastric cancer cells

**Figure 2:**
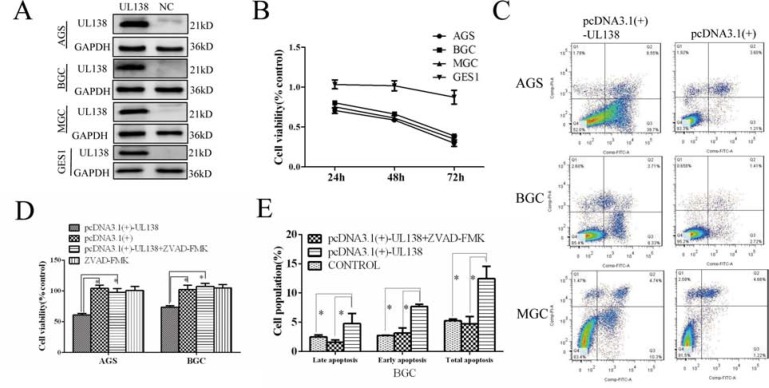
Overexpression of pUL138 inhibits cell viability and induced apoptosis in different gastric cancer cell lines (**A**) Cells transfected with pcDNA3.1(+)-UL138 plasmids (UL138) and pcDNA3.1(+) plasmids (NC) were detected by Western blot at 48 hr post transfection. (**B**) Relative cell viability of GC cells when transfected with pcDNA3.1(+)-UL138 compared with pcDNA3.1(+). Cell proliferation was measured at indicated times post transfection. (**C**) Apoptosis assay by flow cytometry with annexin V-FITC/PI double-staining. GC cells transfected with pcDNA3.1(+)-UL138 present larger population of apoptosis compared with pcDNA3.1(+) at 48 hr post transfection. The dual parameter fluorescent dot plots were sorted as viable cells in the lower left quadrant, and apoptotic cells in the right quadrant. (**D**) UL138-caused inhibition of gastric cancer cells was reversed by a broad-spectrum caspase inhibitor z-VAD-FMK (ZVAD). AGS and BGC-823 cells were transfected with pcDNA3.1(+)-UL138 or pcDNA3.1(+) and ZVAD was added at the same time. At 48 hr post infection, cell proliferation was counted by a CCK-8 test and normalized by control cells (without transfection). Data was presented as means ± SEM of three independent tests. *P < 0.05. (**E**) BGC-823 cells were transfected with pcDNA3.1(+)-UL138 or pcDNA3.1(+) or none (control) and were prepared for apoptosis assay by flow cytometry at 48 hr post transfection. Data was presented as means ± SEM of three independent experiments. Statistical analyses of the number of apoptotic cells were shown accordingly. *P < 0.05.

To evaluate potential roles of pUL138 (UL138 protein) in gastric cancer development, the recombinant pcDNA3.1(+)-UL138 plasmids expressing UL138 were transiently transfected into normal gastric mucosal epithelial cell line GES-1 and three gastric cancer cell lines AGS, BGC-823 and MGC-803 (Figure 2A and Figure S2A). Our data showed that transfection of pcDNA3.1(+)-UL138 led to significant inhibition on cell viability of gastric cancer cells (41.4%, 33.7%, 38.7% decrease of cell viability in AGS, BGC-823, MGC-803 cells, respectively) but no obvious effect (viable cells decreased by 1.6%) on the growth of normal gastric mucosal epithelial cells after transfection 48 hr. Furthermore, the inhibitory effect of pUL138 on the proliferation of gastric cell lines was in a time-dependent manner (Figure 2B).

We next investigated the effect of pUL138 on GC cell apoptosis. As shown in Figure 2C and Figure S5A, UL138-expressing GC cells showed a significant and constant increase in the apoptotic cell population. At 48 hr post transfection, the apoptosis rate of UL138 vs control groups was 31.40% ± 4.35% versus (vs) 8.29% ± 1.80% in AGS cells, 13.41% ± 1.18% vs 4.68% ± 0.39% in BGC-823 cells, and 10.30% ± 0.97% vs 2.28% ± 0.06% in MGC-803 cells, respectively (*P < 0.05*). The apoptotic change caused by pUL138 overexpression was consistent with the observation of nuclei-staining with Hoechst 33258 by fluorescence microscopy (Figure S5B). In addition, we found that a broad-spectrum caspase inhibitor z-VAD-FMK was able to prevent the death of AGS and BGC-823 cells caused by UL138 overexpression. After z-VAD-FMK (50 μM) treatment for 48 hr, percentage of live AGS and BGC-823 cells normalized to the control cells (100%) was 60.9%, 104.2%, 98.0%, 100.7% and 73.7%, 102.1%, 107.1%, 104.8%, respectively (Figure 2D). Meanwhile, treatment with z-VAD-FMK decreased the apoptotic cell population to the similar extent as the control group (Figure 2E, Figure S5C and Figure S5D).

**Figure 3:**
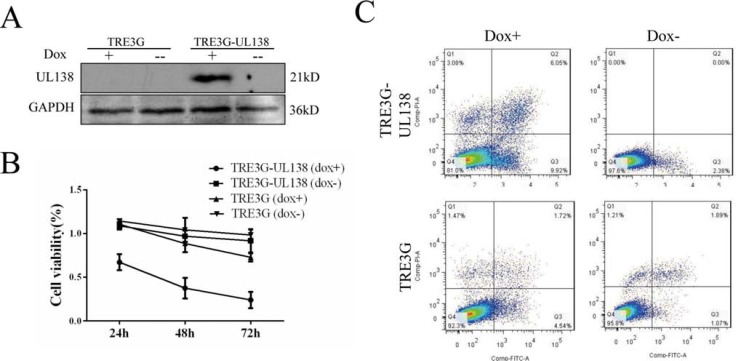
Inductive pUL138 expression in stably UL138-transfected BGC-823 cell validates pro-apoptotic function of pUL138 (**A**) Detection of expression of pUL138 in UL138-transfected BGC-823 cells (TRE3G-UL138) by Western blot after induced with 1 μg/ml doxycycline (dox) for 48 hr. TRE3G means BGC-823 cells containing two plasmids pTRE3G and pCMV-Tet3G (BGC-TRE, cells contained two original plasmids of Tet-on system). TRE3G-UL138 means BGC-823 cells containing two plasmids pTRE3G-UL138 and pCMV-Tet3G (BGC-UL138, cells contained recombinational plasmids inductively expressing pUL138). GAPDH served as a loading control. (**B**) Cell proliferation was measured at indicated times post treating with or without dox (1 μg/ml) by CCK-8. pTRE3G-UL138 and pTRE3G referred to UL138-transfected and vector-transfected BGC-823 cell, respectively. +/−, with or without 1 μg/ml dox in cell culture medium. Proliferation was normalized to BGC-823 cells. (**C**) Apoptosis resulted by dox-induced UL138 expression was measured by flow cytometry after Annexin V-FITC/PI double-staining. Cells were treated with or without dox (+/−) at 1 μg/ml for 48 hr.

To further confirm the above observations, a stably transfected BGC-823 cell with a controllable gene expression of UL138 driven by the Tet-On system was constructed (Figure 3A and Figure S6). After exposed to dox for 48 hr, the viability of BGC-UL138 cells was decreased to 66.3% ± 3.02%, and the inhibition was in a time-dependent manner. In contrast, no obvious change of the cell viability was observed in the absence of dox induction (Figure 3B). Flow cytometry apoptosis assay also showed more apoptosis-related cell death in UL138-expressing cells occurred in the presence of dox (Figure 3C), further suggesting a role of UL138 in suppression of gastric cancer cells.

### UL138 induces apoptosis of gastric cancer cells in association with apoptosis-related proteins

**Figure 4:**
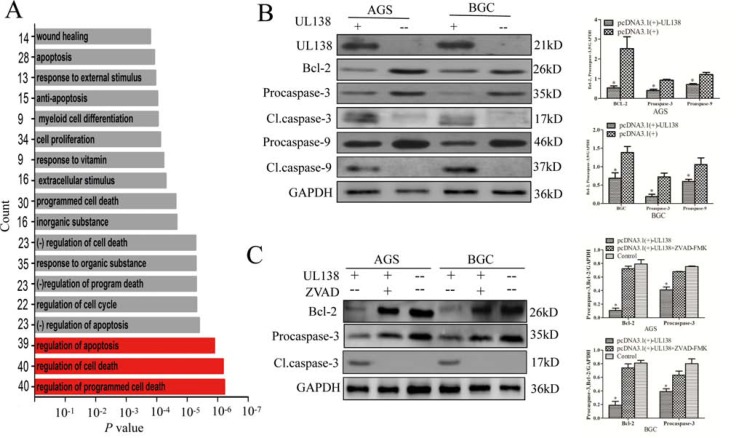
pUL138 is associated with apoptosis-related proteins in gastric cancer cells (**A**) Differentially expressed genes (DEG) between BGC-823 cells transfected with UL138 and control vectors analyzed by mRNA microarray. *P value* histograms of GO terms annotated for the 500 DEGs is as indicated. The length of each bar represents the degree of *P values* obtained by GO analysis. The number of DEGs annotated to each GO is indicated on the left. (**B**) Expressional changes of Bcl-2, caspase-3 and caspase-9 in gastric cancer cells expressing UL138 were determined by Western blotting at 48 hr post UL138 gene transfection. AGS and BGC-823 were transfected with pcDNA3.1(+)-UL138 or pcDNA3.1(+) (indicated as UL138+/−). After 48 hr, the expression of pUL138, Bcl-2, caspase-3 and caspase-9 were determined by Western blotting followed by quantitative densitometric analysis using Image J software. Procaspase indicated caspase precursor and Cl.caspase indicated caspase cleavage. GAPDH served as a loading control. Data are presented as mean +/− SEM of 3 independent experiments. (**C**) z-VAD-FMK treatment reversed effect of UL138 overexpression. Plasmids were transfected as described in (B), then inhibited with or without z-VAD-FMK (ZVAD +/−) and analyzed as described in (B).

To further elucidate the mechanism involved in UL138-mediated apoptosis, the messenger RNA microarray data were analyzed to list differentially expressed gene (DEG) between cells transfected with UL138 and control vector. The biological processes and metabolic pathways of UL138 binding genes were annotated by GO terms with the DAVID program (Supplementary Table S1). The top enriched GO terms in the UL138 interactome were annotated to be involved in regulation of programmed cell death (GO: 0043067), regulation of cell death (GO: 0010941), regulation of apoptosis (GO: 0042981) (Figure 4A). To determine whether UL138 plays a role in several signaling pathways, we superimposed the human Kyoto Encyclopedia of Genes and Genomes pathway database on the UL138 interactome. As shown in Table S1, plenty of identified UL138-associated genes were involved in signaling pathways responsible for the control of key physiological and pathological processes, such as Hematopoietic cell lineage (hsa04640), Cancer development (hsa05200), p53 signaling pathway (hsa04115), and Cell cycle (hsa04110). As expected, Bcl-2 was greatly decreased under the overexpression of UL138 (Figure 4B, Figure S7A) and the effect were in a time-dependent manner (Figure S7C and S7D). In addition, we also observed a significant increase in activation of caspase-9 and caspase-3 in gastric cancer cells expressing UL138 (Figure 4B). At 48 hr post UL138 expression, the expression of procaspase-3 was significantly decreased, whereas cleaved caspase-3 was increased. Moreover, the similar expression changes were also detected in the casapase-9 protein. However, the expression of caspase-8 was not changed (Figure S7B). As expected, z-VAD-FMK treatment reversed the effect of pUL138 on these apoptotic proteins (Figure 4C). Interestingly, the expression lever of Bcl-2 was also changed. As we know, Bcl-2 and caspase-9 were involved in intrinsic apoptosis pathway while caspase-8 was involved in extrinsic apoptosis pathway and caspase-3 was involved in common apoptosis pathway [28]. Our results suggested that the induction of apoptotic cell death caused by pUL138 probably occurred through the intrinsic apoptosis pathway (also known as mitochondrial pathway).

### Screening of UL138-interacting proteins in inducing apoptosis of gastric cancer cells

To identify molecular mechanism of UL138 as a gastric tumor suppressor, we screened potential UL138-interacting proteins by using a customized protein binding array, which allows high-throughput screening of Alexa-Flour-647-labeled UL138-interacting candidates among the 19394 individual human GST-His6-tagged proteins spotted on the chip [29–32]. Sixty eight UL138-interacting candidates were screened with the fluorescence intensity more than 20% of the maximal signal (Figure S8A and Table S2). Based on potential functions annotated by the GO software, these 68 proteins were classified to be associated with cell death/survival, DNA damage/signaling, chromatin remodeling/gene regulation, cancers (Supplementary Table S3). To further narrow the range of the potential UL138-interacting proteins, the PANTHER classification system was used. These 68 proteins could be classified into 12 biological process groups and 21 PANTHER signaling pathway groups accordingly (Figure S8B and S8C). The largest group within the UL138 interactome is the metabolic process family (29.2%). Other significant functional categories included Apoptosis process, Response to stimulus and Immune system processes. Particularly, six potential UL138-interacting proteins (AKT3, MYLK, NAPIL1, HSPA1L, DAXX and DIABLO) were finally chosen in the pathway of apoptosis process. Interestingly, most of these functional groups among the potential UL138-interacting proteins were related with HSPA1L protein, a member of the heat shock protein 70 (HSP70) family. It has been reported that HSP70 has critical role in cancer initiation and progression [33–35]. Therefore, we explored the interaction between pUL138 and HSP70.

**Figure 5:**
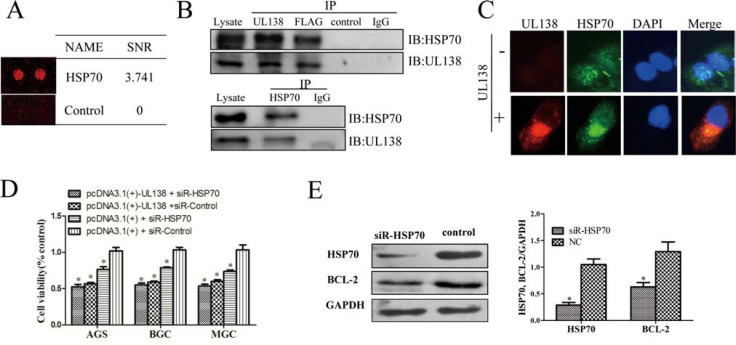
pUL138 interacts with HSP70 protein and blocks its function (**A**) The interaction between UL138 and HSP70 were screened by a human proteome microarray on pure UL138 protein. SNR indicated the average ratio of signal to noise calculated from two duplicated spots. (**B**) HSP70 forms a complex with UL138 protein detected in either UL138 or HSP70 immunoprecipitation (IP). BGC-823 cells were transiently transfected with pcDNA3.1(+)-UL138 tagged with Flag for 48 hr. Protein lysates were assayed for protein-protein interactions by IP using the specific antibodies against the UL138, Flag, or HSP70, and interacting partners were detected by immunoblotting (IB) with antibodies specific to the another protein in the pair, as indicated beside the blot. The pair protein examined in each panel is indicated above the immunoblotting. In each panel, lysates incubated with normal IgG or control serum (as negative control to UL138 specific antibody) served as negative controls. The immunoprecipitation assays are representative of 3 independent experiments. (**C**) Analysis of HSP70 and UL138 co-localization. BGC-823 were seeded on glass coverslips and transfected with pcDNA3.1(+)-UL138 or pcDNA3.1(+) and then incubated for 48 hr. Cells were stained with mouse antibody to pUL138 and rabbit antibody to HSP70. Nuclei were counterstained with DAPI. (**D**) Inhibition of UL138 in cell viability was verified by siRNA-HSP70. Cell viabilities of GC cells at 48 hr post transfection with pcDNA3.1(+)-UL138 (or pcDNA3.1(+)) and HSP70 siRNA (or non-function siRNA (siR-control)) by CCK-8 kits. Results were presented as the mean ± SEM of three individual experiments in each group. (**E**) The protein expression level of Bcl-2 was decreased when transfected with siRNA-HSP70 in BGC cell. Total protein was prepared 48 hr after transfected with HSP70 siRNA (siR-HSP70) or control siRNA (NC). HSP70 and Bcl-2 expressions were analyzed by Western blot followed by quantitated densitometric analysis using ImageJ software. GAPDH served as a loading control.

Co-immunoprecipitation (Co-IP) assay was carried out to validate the direct interaction between HSP70 and pUL138. As shown in Figure 5B, endogenously expressed HSP70 protein was Co-IP in the UL138 complex immunoprecipitated with anti-UL138 or Flag-tag antibody. As expected, no detectable signals of HSP70 were observed in a normal IgG and control sera. Furthermore, reciprocal Co-IP confirmed this interaction by using anti-HSP70 for immunoprecipitation. The *in situ* interaction of UL138 and HSP70 was also evaluated by immunofluorescence microscopy, indicating a co-localization of HSP70 and UL138 in gastric cancer cells (Figure 5C). Similar with the UL138 overexpression, the down-regulation of HSP70 in GC cells significantly inhibited the cell proliferation. At 48 hr after HSP70 siRNA transfection, cell viability in AGS, BGC-823, MGC-803 cell was 76.3%, 78.7% and 73.8%, respectively (Figure 5D). At the same time, the expression level of Bcl-2 was then decreased consequently (Figure 5E). IHC analysis of HSP70 expression in 20 tissues of gastric cancers and adjacent normal tissues indicated that the expression level of HSP70 in gastric adenocarcinoma tissues was significantly higher than that in paired normal gastric tissues (Figure S9B). In addition, up-regulation of HSP70 in tumor tissues was also associated with differentiation of gastric cancer (Figure S9B). These data further confirmed the relationship between UL138 and HSP70.

However, compared with the control, the expression level of HSP70 did not change in UL138-expressing GC cells (Figure S9A). In addition, there was no significant difference in cell death between cells overexpress UL138 only and those combined with HSP70 down-regulation using siRNA (Figure 5D). So, we speculated that blocking the function of HSP70 was the partial mechanism in pUL138-inducing apoptosis process.

### UL138 overexpression efficiently suppresses human tumor growth *in vivo*

**Figure 6:**
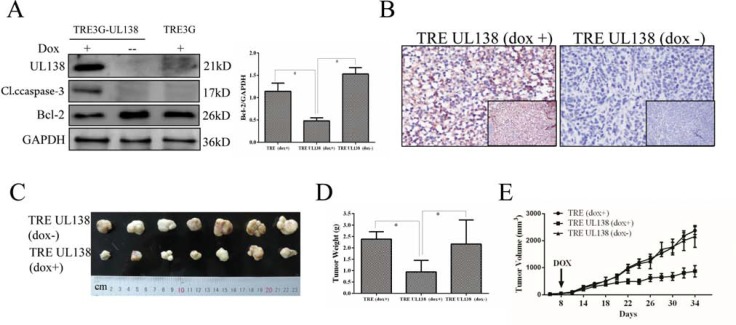
Expression of pUL138 in gastric cancer cells inhibits tumor growth *in vivo* (**A**) The expression of UL138, caspase-3 and Bcl-2 in tumor tissue was examined by Western blotting. TRE3G-UL138 and TRE3G referred to the xenograft tumors from the stably UL138-transfected and vector-transfected BGC-823 cells, respectively. +/−, with or without dox induce. GAPDH served as a loading control. *P < 0.05. (**B**) Immunohistochemical staining of the UL138 protein in the xenograft tissue sections of nude mice subcutaneously injected with BGC-823 stable cell lines and subsequent induced by dox (magnification, × 100 and × 400). The mice without dox used as control (magnification, × 100 and × 400). (**C**) Two groups of dissected tumors were photographed. TRE-UL138 (dox+) was group A and TRE-UL138 (dox-) was group B mentioned in Materials and Methods. (**D**) At the end of the xenograft model experiment, dissected tumors were weighted. TRE-UL138 (dox+) was group A. TRE-UL138 (dox-) was group B. TRE (dox+) was Group C. Group A, B, C were mentioned in Materials and Methods. *P value* was obtained by using a two-tailed Student's *t*-test. *P < 0.05. (**E**) During the xenograft model experiment, tumor volumes were measured in every other day. The indicated arrow meaned the day when dox was added into water for specific groups. Results are presented as the mean ± SEM of 7 mice in each group. *P < 0.05.

To further evaluate the potential role of UL138 in cancer repression *in vivo*, we did subcutaneous transplantation of stably UL138-transfected BGC-823 cells in nude mice and established a xenograft animal model. Based on whether added dox which was required for conditional expression of UL138 in stably UL138-transfected BGC-823 cells to their drinking water, these mice were devided into two groups (dox+ group and dox- group). UL138 protein was showed present in tumor tissues in the animals of dox+ group by Wentern blot analysis, but absence in the group of dox- (Figure 6A). IHC results also verified the successfully induced expression of pUL138 *in* *vivo* compared with control groups (Figure 6B). During 34 days, tumor growth was observed by measuring the tumor size every other day. As shown in Figure 6C, 6D and 6E, tumor weights and volumes in dox+ group were significantly less than those in dox- group (*P < 0.05*). Our data indicated that pUL138 expression greatly suppressed subcutaneous gastric cancer growth. To explore the tumor suppression mechanism mediated by UL138-inducing apoptosis, caspase-3 and Bcl-2 expressions in xenograft tumor tissues was further detected by Western blotting. As shown in Figure 6A, the expression of caspase-3 and Bcl-2 were regulated simultaneously in the tumor tissues of UL138 expression, indicating that Tet-On system-controlled UL138 expression in the xenograft animal model induced tumor cell death by apoptosis.

## DISCUSSION

Emerging data implicated that cytomegalovirus may be associated with some malignant diseases such as glioblastmas, medulloblastomas, colorectal and prostatic cancers [3, 5, 36, 37]. Although, there was no direct evidence considering HCMV as oncogenic virus, several studies recently reported that some HCMV encoded genes, such as IE, US28, UL36–38, UL97 and so on, took part in cellular transformation associated with tumor development [38]. Besides, several HCMV genes play roles in cell cycle, apoptosis, invasion and migration of cancer cells and tumor angiogenesis [6, 39–45]. However, our work provides a completely new insight of the viral protein UL138 in suppression of cancer growth.

As a multifunctional HCMV protein, pUL138 is well-known in a requirement of efficient latency of virus infection and anchored in Golgi membranes with a large C-terminal tail in cytoplasm, suggesting its potential functions on protein trafficking, stress, or apoptosis through the interaction with other molecules [46, 47]. Activation of UL138 results in up-regulation of tumor necrosis factor receptor 1 (TNFR1) [25, 26], whereas a dramatic loss of multidrug resistance–associated protein-1 (MRP1) [27] on the surface of infected host cells. Our previous study demonstrated that UL138 was detected in most of human tumor tissues [24]. However, the specific function of UL138 in the development of gastric cancer remains unknown.

In our study, we demonstrated that UL138 was down-regulated in gastric adenocarcinoma tissues, and even lower in poorly or none differentiated tumors in gastric cancer patients. Overexpression of UL138 obviously inhibited GC cells proliferation and induced apoptosis. More importantly, overexpression of UL138 efficiently leads to suppression of human tumor growth in a xenograft animal model of gastric cancer. Interestingly, this UL138-directed cell apoptosis does not occur in normal gastric mucosal cells, indicating a particular suppression of the viral protein UL138 on the development of gastric cancer.

Additionally, our study demonstrated that UL138 could interact with multiple host proteins during its induction of apoptotic cell death in the protein-binding microarray assay. Both GO and PANTHER analyses showed that the screened UL138-interacting proteins are involved in different biological events such as metabolic process, apoptosis process, immune responses, DNA damage, chromatin remodeling, and oncogenes. This is also consistent with the previously reports, a stress-inducible strategy of translation initiation stimulates expression of UL138 under a variety of cellular contexts [48], and UL138 interaction with different host proteins would contribute to distinct functions of this protein in multiple cell types [21]. Combined with Co-IP and immunostaining, we discovered that HSP70 was one of UL138-interacting proteins.

It has been demonstrated that overexpression of HSP70 in different types of cancer cells has been correlated to poor differentiation of tumors, lymph node metastasis and a shorter patient survival prognosis [33–35]. Besides, expression of HSP70 in early stage of gastric adenocarcinoma was enhanced, suggesting its function on histopathological differentiation of gastric cancer [49–51]. Clinicopathological study of HSP70 further indicated that the HSP70 expression was inversely related to poor differentiation or diffuse types of tumor cells [52]. Recently, Wang W, *etc* [53] constructed an AdSurp-HSP70 viral therapy system for gastric cancer targeted immunotherapy. Our data also showed more HSP70 expressed in tumors compared with adjacent normal tissues.

It has been known that development of malignant tumor may depend on the dynamic balance of cell proliferation and apoptosis while cancer cells are exposed to various stresses. Normally, HSP70 and its co-chaperone BAG3 rescue cells from apoptosis by stabilizing anti-apoptotic Bcl-2 family proteins and inhibiting caspase-3 cleavage [54–58]. Our data also indicated that inhibition of HSP70 in GC cells by siRNA-HSP70 could induce apoptotic cell death. Furthermore, the expression level of Bcl-2 was then decreased consequently, as the same phenomenon of UL138 function, further confirmed the relationship between UL138 and HSP70.

**Figure 7:**
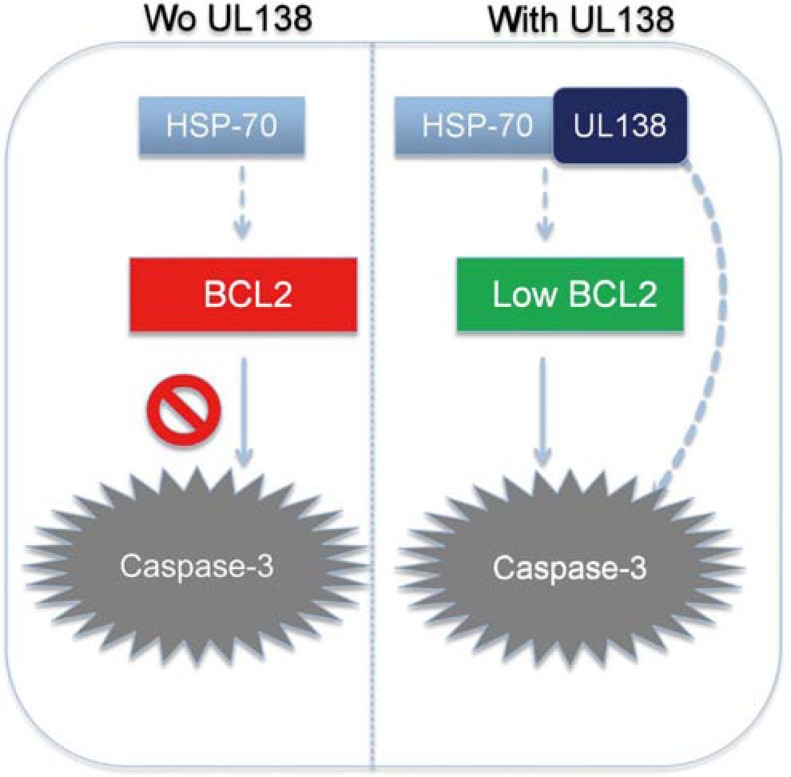
Potential mechanism of pUL138 inducing GC cells apoptosis Normally, HSP70 rescue cells from apoptosis by stabilizing anti-apoptotic Bcl-2 family proteins and inhibiting caspase-3 cleavage. pUL138 expression in GC cells can directly bind to and block HSP70 function, along with several other apoptosis-related proteins. This binding not only causes degradation of Bcl-2, but also inactivates caspase-associated apoptotic cancer cell death.

However, pUL138 showed significantly higher effect than HSP70-siRNA treatment in inducing apoptosis of GC cells. In addition, HSP70 down-regulation by siRNA did not increase the cell death induced by UL138 overexpression (Figure 5D), suggesting another proteins and pathways were associated with pUL138-inducing apoptosis in GC cells. So, we speculated that the blocking of HSP70 function was the partial mechanism in pUL138-inducing apoptosis process (Figure 7).

Taken together, we demonstrate for the first time that the cytomegalovirus protein UL138 could act as anti-oncogene and specifically induce apoptosis of gastric cancer cells by partially interacting with HSP70 and subsequently decreasing Bcl-2 and inducing caspase-3 cleavage. Our findings will improve the understanding of the cytomegalovirus viral genes in the development of gastric cancer, and might provide a novel target for the treatment of gastric cancer.

## MATERIALS AND METHODS

### Patients and specimens

Forty-nine GC patients with HCMV positive infection in the cancer tissues were enrolled in this cohort study (Figure S1). These patients were diagnosed as gastric cancer by endoscopic biopsy, and were admitted for surgical treatment from March 2014 to September 2014 in the First Affiliated Hospital of Wenzhou Medical University (Zhejiang Province, China). Paired specimens of gastric cancer tissues and the corresponding adjacent normal gastric tissues were obtained from these patients. The HCMV positive infection in the tissues were verified by PCR detection of UL138 DNA [24]. None of the patient had pre-operative radiation or chemotherapy but followed strict post-surgical chemotherapy according to the NCCN gastric cancer guidelines. After operation, gastric adenocarcinoma was histopathologically diagnosed and confirmed by the Pathology Department according to the criteria of the World Health Organization. The information of patients is listed in Table 1. Each patient was informed by written consent form and the study was done after the approval of the Human Research Ethics Committee at the First Affiliated Hospital of Wenzhou Medical University.

### Cells and animal

Gastric cancer cell of AGS was obtained from the American Type Culture Collection (ATCC^®^, Manassas, VA, USA). Gastric cancer cells of MGC-803, BGC-823 and Human Gastric Mucosal Epithelial Cell Line (GES-1) were obtained from the Type Culture Collection of Chinese Academy of Sciences (Shanghai Institute of Biochemistry and Cell Biology^®^, Shanghai, China). All these cells were cultured in 37°C at a humidified 5% CO_2_ atmosphere. Female nude mice (4–5 week old, BALB/c) were purchased from the shanghai SLAC laboratory animal CO. LTD (Shanghai, China) and rose under specific pathogen-free (SPF) condition. All animal tests and experimental procedures were approved by the Ethical Committee of Wenzhou Medical University and Laboratory Animal Management Committee of Zhejiang Province (Approval ID: wydw2015–0146).

### Quantitative real-time PCR (RT-qPCR)

Total RNA was extracted by using the TRIzol reagent (Invitrogen Life Technologies^®^, Grand Island, USA) according to the manufacturer's protocol. Potential contamination of DNA in the purified RNA was avoided by using a DNAfree reagent (Ambion, Carlsbad, USA). First-strand cDNA was reverse transcribed with ReverTra Ace^®^ qPCR RT kit (Toyobo^®^, Tokyo, Japan) from 1 μg of total RNA. The detection of UL138 RNA was assessed by real-time PCR with RNA-direct^™^ SYBR^®^ Green Real-time PCR Master Mix (Toyobo) and specific primers (forward, 5′-GGCACGACACCTTCAAAC-3′; and reverse, 5′-AGA CACTTCCTCCCAACG-3′; 200 bp). GAPDH was chosen as internal control gene (forward, 5′-CAGGGCTGCTTT TAACTCTGGTAA-3′; and reverse, 5′-GGGTGGAAT CATATTGGAACATGT-3′; 101 bp). In CFX96 Touch^™^ Real-Time PCR Detection System (BioRad), PCR cycles involved at 95°C for 5 min; then followed by 40 amplification cycles of 94°C for 30 sec, 57°C for 30 sec, 72°C for 30 sec. Melting curves were generated for each real-time PCR to verify the specificity of each PCR reaction. Duplication was performed in real-time PCR for accuracy judgment.

### Immunohistochemistry (IHC)

IHC staining for UL138 using anti-UL138 polyclonal mouse antibodies that make by our experiment. Paired formalin-fixed and paraffinembedded tissue blocks (*n =* 49) from gastric adenocarcinoma and normal resection margin were cut into 5 μm sections and adhered to 0.1% poly-L-Lysine treated glass slides (Maixin-Bio^®^, Fuzhou, Fujian Province, China) and dewaxed using dimethyl benzene followed by immersion in distilled water. Endogenous peroxidase activity was inhibited by incubation in a 0.3% hydrogen peroxide bath for 10 min followed by three washes with PBS (pH 7.4). Antigen retrieval was carried out by high-pressure antigen retrieval for 2 min in citrate antigen retrieval solution (pH 6.0, Maixin-Bio). After cooling to room temperature, slides were washed three times by 0.01 mol/l phosphate buffer solution (PBS, pH 7.4).

For the detection of UL138, the sections were incubated in 1:1000 anti-UL138 polyclonal mouse antibodies in room temperature for 3 h. For the detection of HSP70, the sections were incubated in primary anti-HSP70 antibody (Abcam, Cambridge, USA) diluted to 1:1000 in PBS, in a humidified chamber at 4°C overnight. Sections were then incubated in horseradish peroxidase (HRP)-conjugated secondary goat anti-mouse antibodies (MaiXin Bio, Fuzhou, China) at 37°C for 30 min. The color reaction was developed using the DAB kit (Zhongshan Golden Bridge Bio^®^, Beijing, China) for 5 min according to the manufacturer's protocol. Specimens were counterstained with hematoxylin, rinsed in PBS, dehydrated through graded ethanol (80, 90 and 100%) and by dimethylbenzene. The obtained images were subsequently processed using Image-Pro Plus software (Media Cybernetics, Rockville, MD, USA).

### *In situ* hybridization

*In situ* hybridization was further used to confirm the expression of UL138 in tissues. The specific probe strictly matched with UL138 nucleotide sequence was designed by the Oligo 7.0 software. The probe was modified by locked nucleic acid (LNA) (5′-atCgtGgcCatTctCtgCtaTctG-3′, capital: LNA, lowercase: DNA) and labeled with digoxigenin (DLG) at the 5′-end, and synthetized in the Takara Bio Inc. A probe (5′-atCgtCgcGatActCtgAtaTctC-3′, capital: LNA, lowercase: DNA) containing three mismatches was set as negative control. Tissues fixed in 4% paraformaldehyde and embedded in paraffin were cut into 5 μm sections and picked on 0.1% poly-L-Lysine treated glass slides. After dehydrated in a graded series of ethanol baths, the sections were treated for 5 min with proteinase K (10 μg/ml) and refixed in 4% paraformaldehyde for 10 min. After washed twice in PBS, the sections were prehybridized for 2 hr in hybridization buffer (Wuhan Boster bio-engineering company, Wuhan, China). The section were then hybridized overnight in the hybridization solution containing 50 nM DLG-labeled probe at 42°C. Slides were then washed twice in 2 × SSC and twice in 0. × SSC at 50°C. Immunological detection was carried out with the anti-digoxigenin Fab conjugated to alkaline phosphatase (Roche, Mannheim, Germany) according to the manufacturer's instructions.

### Recombinant plasmid vector expressing UL138 and cells transfection

In our previously experiment, the recombinant UL138 expression plasmid has successfully been constructed. The sequence of UL138 open reading frame (ORF) tagged with Flag was subcloned into pcDNA3.1(+) vector by ligating into the BamHI/XhoI sites. The recombinant plasmid was sequenced to confirm the successful construction. The plasmid were transfected into gastric cancer cells of AGS, BGC-823, MGC-803 and Human Gastric Mucosal Epithelial Cell Line (GES-1) with lipofectamine 2000 (Life Technologies, Bethesda, MD) according to the manufacturer's protocol. The expression of UL138 in these transfected cells were confirmed by anti-UL138 and anti-flag specific antibodies.

### Stably transfected BGC-823 cell with a controllable gene expression of UL138 driven by the Tet-On system

The Tet-On 3G Systems are the inducible gene expression systems for a gene of interest (GOI) under the control of a TRE3G promoter in mammalian cells. In the presence of doxycycline (dox), cells stably transfected with the Tet-On system will expresses high levels of GOI. To construct the stably transfected BGC-823 cell with a controllable gene expression of UL138 driven by the Tet-On system, the encoding sequence of UL138 was first inserted into pTRE3G expression vector (Clontech, CA) by ligating into the BamHI/EcoRV sites under the control of pTRE. The constructs were designated as pTRE3G-UL138. The plasmid pTRE3G-Luc encoding luciferase was used as control. BGC-823 cells were transfected with pCMV-Tet3G plasmid and selected with G418 (Invitrogen, Carlsbad, CA, United States) for 3–4 week to generate a stable cell line that constitutively expressing Tet-On 3G transactivator. Then these cells were screened and the highest levels of induction cell lines were chosen. pTRE3G-UL138 expression vector was then transfected into the Tet-On 3G stable cell line and screened with hygromycin. A double-stable cell line BGC-823/pTRE3G-UL138 was established after second round of drug selection. The expression of UL138 in the BGC-823/pTRE3G-UL138 cells in the presence and absence of 1 μg/ml dox were confirmed by anti-UL138 specific antibodies (Figure S6B).

### Cell viability

Cell viability was evaluated by Cell Counting Kit-8 (CCK-8, Dojindo, Kumamoto, Japan). AGS, BGC-823, MGC-803 and GES-1 cells transfected with pcDNA3.1(+) plasmid vector expressing UL138 were seeded in 100 μl PRMI-1640 medium at 5 × 10^3^ cells/well in 96 well plates. The transfection of blank pcDNA3.1(+) plasmid vector was set as control. After cells were cultured for 24 hr, 48 hr and 72 hr, respectively, 10 μl CCK-8 was added and continue culture for 1.5 hr at 37°C. The absorbance in 450 nm were measured by Microplate Reader (Thermo, United States). To evaluate the cell viability of BGC-823 cells stably transfected with a controllable gene expression of UL138, cells were first induced in the presence and absence of 1 μg/ml dox for 24 hr, 48 hr and 72 hr, respectively. For the inhibitory experiment, z-VAD-FMK (ZVAD) was co-incubation with UL138-expressing plasmid in the culture medium.

### Apoptosis analysis by flow cytometry

Cells were seeded at 1 × 10^5^ cells per well in six-well plates. BGC-823 cells stably transfected with a controllable gene expression of UL138 were induced in the presence or absence of 1 μg/ml dox for 24 hr, 48 hr and 72 hr, respectively. AGS, BGC-823 and MGC-803cells transfected with pcDNA3.1(+) recombinant plasmid encoding UL138 were harvested at 24 hr, 48 hr and 72 hr, respectively. These cells were incubated with 10 μl/mL PI and 10 μl/mL Annexin V-FITC according to the manufacturer's instructions of FITC-Annexin V Apoptosis Detection Kit (BD, San Diego, USA). BD FACS Calibur™ Flow Cytometer was used to analyze these cells. The data analysis was done by ModFit Software. ZVAD was also used in the inhibitory experiment, added to the culture medium after plasmid transfection.

### Hoechst 33258 assay

Hoechst 33258 was used to determine the nuclear fragmentation by staining the apoptotic nuclei. AGS, BGC-823, MGC-803 and GES-1 cells transfected with pcDNA3.1(+) recombinant plasmid encoding UL138 for 48 hr were used for Hoechst 33258 assay. Cells were washed in cold PBS and fixed with 4% paraformaldehyde for 10 min at 37°C. The fixed cells were then stained with Hoechst 33258 for 10 min. After repeatedly washing with PBS, the cells were examined under a fluorescence microscope (Nikon C1–i, Japan).

### Immunoblotting

Total proteins of transfected AGS, BGC-823, MGC-803 and GES-1 cells were extracted using protein lysis buffer (Beyotime Institute of Biotechnology, Beijing, China) supplemented with protease inhibitor cocktail (Pierce) at 4°C for 30 min. The concentrations of these cell total proteins were determined using the BCA assay kit. Protein samples (20 μg/lane) were separated using 8–12% SDS-PAGE and then electrophoretically transferred to polyvinylidene difluoride membranes (Millipore, MA, USA). After blocking with 5% skim milk for 1.5 hr at 37°C, the membranes were then incubated with the primary antibodies at 4°C overnight, the antibody of caspase-3, caspase-9, Bcl-2 (Cell Signaling Technology, Beverly, MA, USA), GAPDH (Santa Cruz, CA, United States) and HSP70 were diluted with TBST in the concentration of 1:1000, the antibody of UL138 was diluted to 1:2000. The membranes were then washed with TBST buffer for 3 × 5 minutes and incubated with the secondary antibodies (HRP-conjugated goat anti-rabbit IgG (Beyotime Institute of Biotechnology, Beijing, China) for 2 hr at room temperature. The bands were detected using enhanced chemiluminescence and visualised by a Gel Doc 2000 (BioRad, USA) and the data was analyzed by Image J.

### Immunofluorescent localization

Co-localization of cellular proteins in transfected cells were observed by immunofluorescence. Briefly, cells (5 × 10^4^ cells/well in 6-well plate) were mock transfected or transfected with recombinant plasmid encoding pUL138 for 48 hr. AGS, BGC-823 and MGC-803 cells were fixed in 4% paraformaldehyde at room temperature for 10 min, and permeabilized by 0.3% Triton X-100 at room temperature for 10 min. Then cells were blocked in RPMI-1640 supplemented with 10% FBS for 60 min at 37°C. Gastric cancer cells were double-stained with the primary antibodies include a mouse antibody specific to pUL138 and a rabbit antibody specific to HSP70, respectively. Secondary antibodies conjugated to fluorescent molecules included Cy^™^ 3-conjugated goat anti-mouse and FITC-conjugated goat anti-rabbit IgG (Invitrogen). Secondary antibodies were applied in RPMI-1640 for 1 h at 37°C. Cells then were incubated in 1 ug/ml DAPI (4′, 6′-diamidino-2-phenylindole) for 10 min at room temperature. Coverslips were mounted on slides and visualized using a fluorescence microscope (Nikon C1–i, Japan). Images were recolored artificially.

### RNA extraction and microarray

Total RNA of the cells were transfected with UL138 and the control vectors were extracted by Trizol, and then purified with magnetic beads of Agencourt Ampure (APN 000132, Beckman Coulter). RNA target preparation for microarray processing was carried out according to the GeneChip^®^ 3′ IVT Express Kit. The data were analyzed with Robust Multichip Analysis (RMA) algorithm using default analysis settings and global scaling by Partek^®^ Genomics Suite 6.6. Values presented are log2 RMA signal intensity. Data normality analysis was done by one-way ANOVA to screen out the differential expression gene. Then, the pathways and the processes of major biological significance was determined by the Database for Annotation, Visualization and Integrated Discovery (DAVID) and its importance was based on the Gene Ontology (GO) annotation function and Kyoto Encyclopedia of Genes and Genomes (KEGG) pathway function.

### Protein array

The Johns Hopkins Medical Institutions Protein Microarray Core produced the protein binding microarray chips that with 19394 individual human GST-and His6-tagged full-length proteins. 1.5 μg/μl of protein that recombinant pUL138 purified from BL21/pET21a-UL138 was labeled with Alexa-Flour-647 microscale protein labeling kit (A30009, Molecular Probes/Invitrogen). The data were extracted by GenePix Pro 6.0 from the microarray images. Background was defined as signals less than 20% of the maximal signal and removed from the subsequent analysis. The signal-to-noise ratios (SNR = F635 median/B635 median) were first calculated for all the spots to generate the candidate list of UL138 binding proteins. The SNR of a protein was averaged calculated from two duplicated spots.

### Bioinformatics analysis of the UL138 interactome

PANTHER (Protein Analysis through Evolutionary Relationships) system was used to classify the UL138 interactome, which is a unique resource that classifies genes and proteins according to their functions [59]. To determine whether there was any types of proteins over-expression, enrichment analysis of Gene Ontology (GO) terms [60], Kyoto Encyclopedia of Genes and Genomes pathways [61], and Pfam domain families [62] was performed by using the Web-accessible program DAVID 6.7 [63]. The default human proteome was used as the background list. The statistical significance of the enrichments was evaluated by modified Fisher's exact test (EASE score), and a *P*-value for each term was calculated by applying a Benjamini-Hochberg false discovery rate correction [63]. The GO categories GO SLim Generic assignment, the distribution of cellular components, molecular functions, and biological processes of the UL138 interactome were analyzed.

### Immunoprecipitation

BGC-823 cells in six-well plates were transfected with the recombinant plasmid encoding pUL138 by lipofectamine 2000, following the manufacturer's instructions. 4 μg of expression constructs (the plasmid of pcDNA3.1(+)-UL138 or pcDNA3.1(+)) was mixed in 250 μl of Opti-MEM, and 10 μl of lipofectamine 2000 was mixed in 250 μl Opti-MEM. Complexes were combined and added to plates for a 6 h-incubation before replacing the old growth medium.

For cell immunoprecipitations (IP), cells at 48 hr post transfection, were lysed for 30 min on ice with IP buffer (Pierce, Rockford, IL). Lysates were centrifuged to clear nuclear debris at 12, 000 g for 10 min at 4°C. 100 μg of the cell supernatant lysate was mixed with 1 μg of mouse antibodies specific to pUL138, anti-flag and anti-HSP70, respectively. Then we according to the manufacturer's protocol of pierce classic IP kit, the immune complexes were collected with elution buffer by centrifugation at 3000 g for 1 min at 4°C, resuspended in reducing SDS sample buffer, heated at 95°C for 10 min, and analyzed by immunoblotting.

### Assessment of UL138 function in gastric cancer xenograft nude mice model

To assess the tumoricidal activity of UL138, nude mice were used to establish gastric cancer xenograft animal model by injecting subcutaneously 1 × 10^7^ BGC-823/pTRE3G-UL138 cells (stably transfected with a controllable gene expression of UL138 driven by the Tet-On system) into mouse armpit. The animals were divided into two groups (*n =* 7) when tumor appeared after cells injection for 8 days. In group A (dox+), dox with 1 mg/ml concentration was added into the mouse drinking water to induce the expression of UL138. In group B (dox-), the animals were given drinking water without dox. As control, seven nude mice injecting subcutaneously 1 × 10^7^ BGC-823/pTRE3G cells with no UL138 gene were given drinking water with dox (group C). Tumor growth was recorded every other day for 34 days by measuring the two dimensional longest axis (a) and shortest axis (b) with a caliper. The tumor volume was calculated using the following formula: volume in mm^3^ = (3.14ab^2^)/6. At the end of experiment, the animals were been sacrificed, the tissues of tumor were collected for further analysis.

### Statistical analyses

All statistical results of the data were analyzed by SPSS16.0. The data were reported as the mean ± SEM, ANOVA and student's *t* test for repeated data was performed for the differences between the groups. *P* < 0.05 was defined to be statistically significant.

## SUPPLEMENTARY FIGURES AND TABLES




