# Supplementary material for: The cytomegalovirus protein UL138 induces apoptosis of gastric cancer cells by binding to heat shock protein 70
Source: Oncotarget. 2015 Dec 30;7(5):5630–45. doi: 10.18632/oncotarget.6800 (PMC4868710; doi:10.18632/oncotarget.6800)
Supplement: Supplementary file 1 [file oncotarget-07-5630-s001.pdf]

The cytomegalovirus protein UL138 induces apoptosis of gastric cancer cells by binding to heat shock protein 70

Supplementary Materials

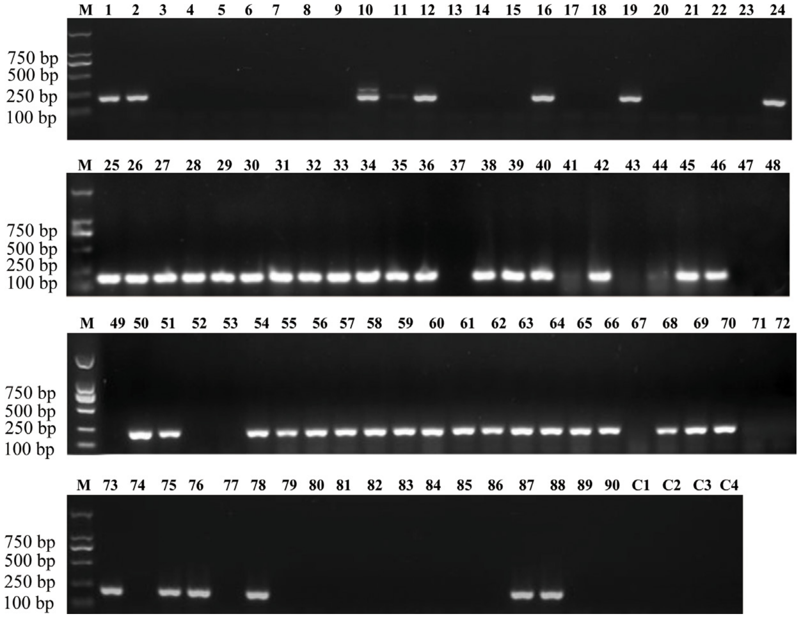

Supplementary Figure S1: UL138 DNA detection in gastric tumor tissues and cells by PCR method. Lane 1-90, 90 tumor tissues from the patients with gastric cancer; C1-C4, AGS, BGC-823, MGC-803 and GES-1 cells, respectively.

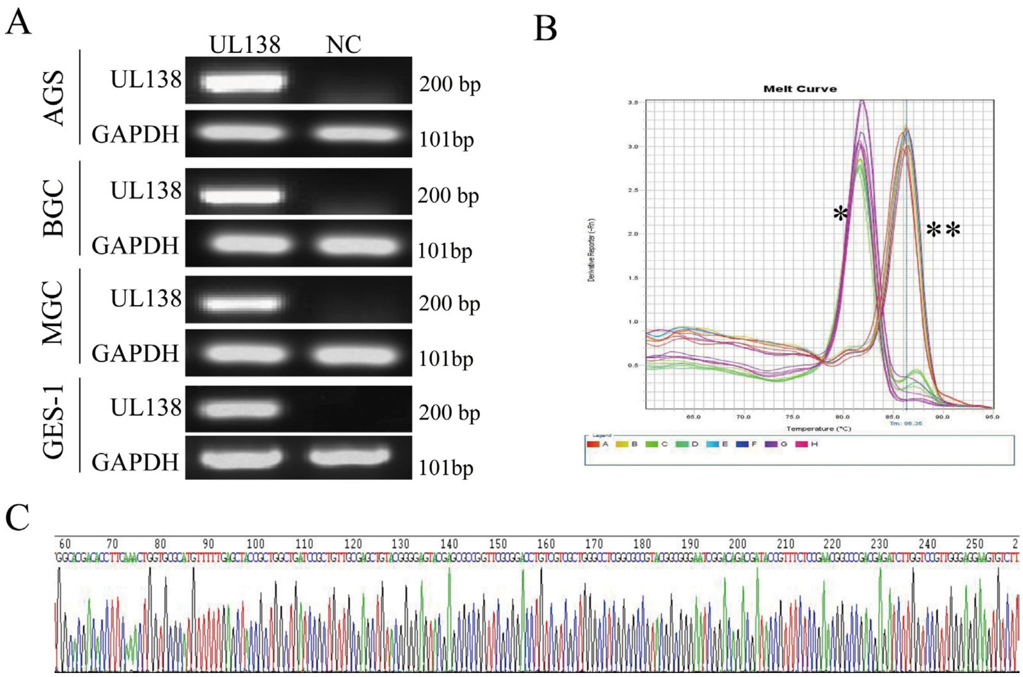

Supplementary Figure S2: The specificity of UL138 primers in the RT-qPCR assays. (**A**) The RNA of cells which was transfected with pcDNA3.1(+)-UL138 plasmids (UL138) or pcDNA3.1(+) plasmids (NC) were detected by RT-PCR at 48 hr post transfection. (**B**) Melting curve; *UL138; **hGAPDH. (**C**) Sequencing results of UL138 PCR product.

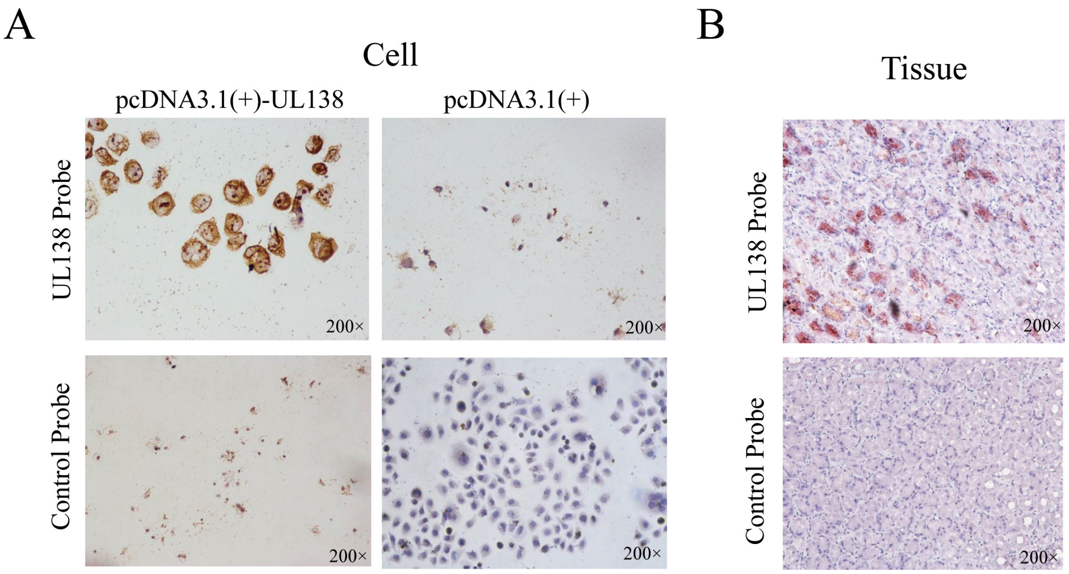

Supplementary Figure S3: The specificity of UL138 probe in the ISH assays. (**A**) Cells transfected with pcDNA3.1(+)-UL138 plasmids (left row) and pcDNA3.1(+) control plasmids (right row) were detected by UL138-based ISH at 48 hr post transfection. The probe containing three nucleotide mutation was set as control. The magnification was ×200. (**B**) The detection of UL138 transcript in gastric tissues by UL138-specific probe and control. The magnification was ×200.

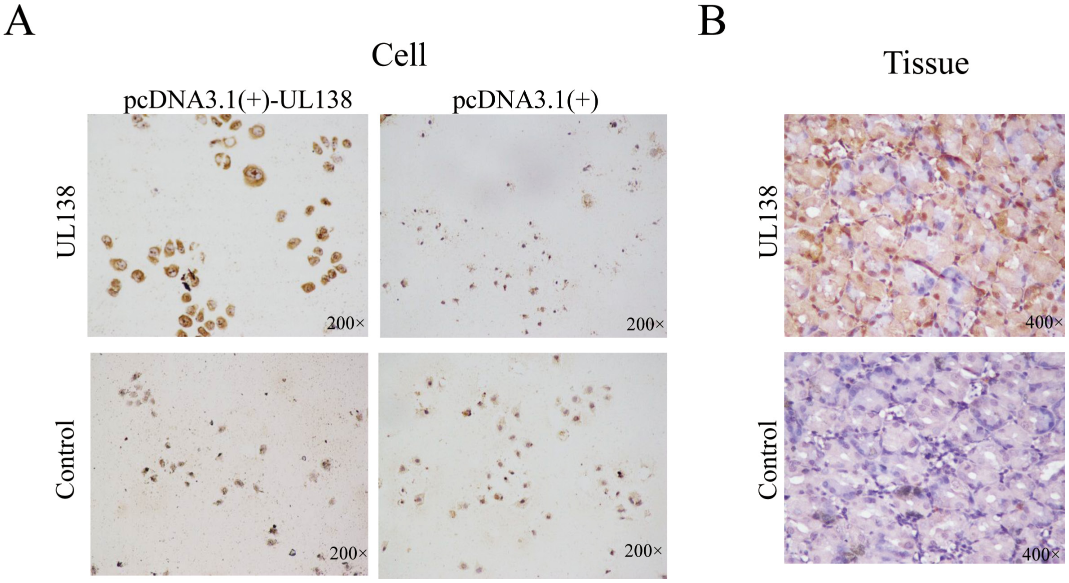

Supplementary Figure S4: The specificity of UL138 antibody in the IHC assays. (**A**) Cells transfected with pcDNA3.1(+)-UL138 plasmids (left) and pcDNA3.1(+) plasmids (right) were detected by UL138-specific antibody using IHC at 48 hr post transfection. Pre-immune sera were set as control (bottom). The magnification was ×200. (**B**) The detection of UL138 protein in gastric tissues by UL138-specific antibody and control sera. The magnification was ×400.

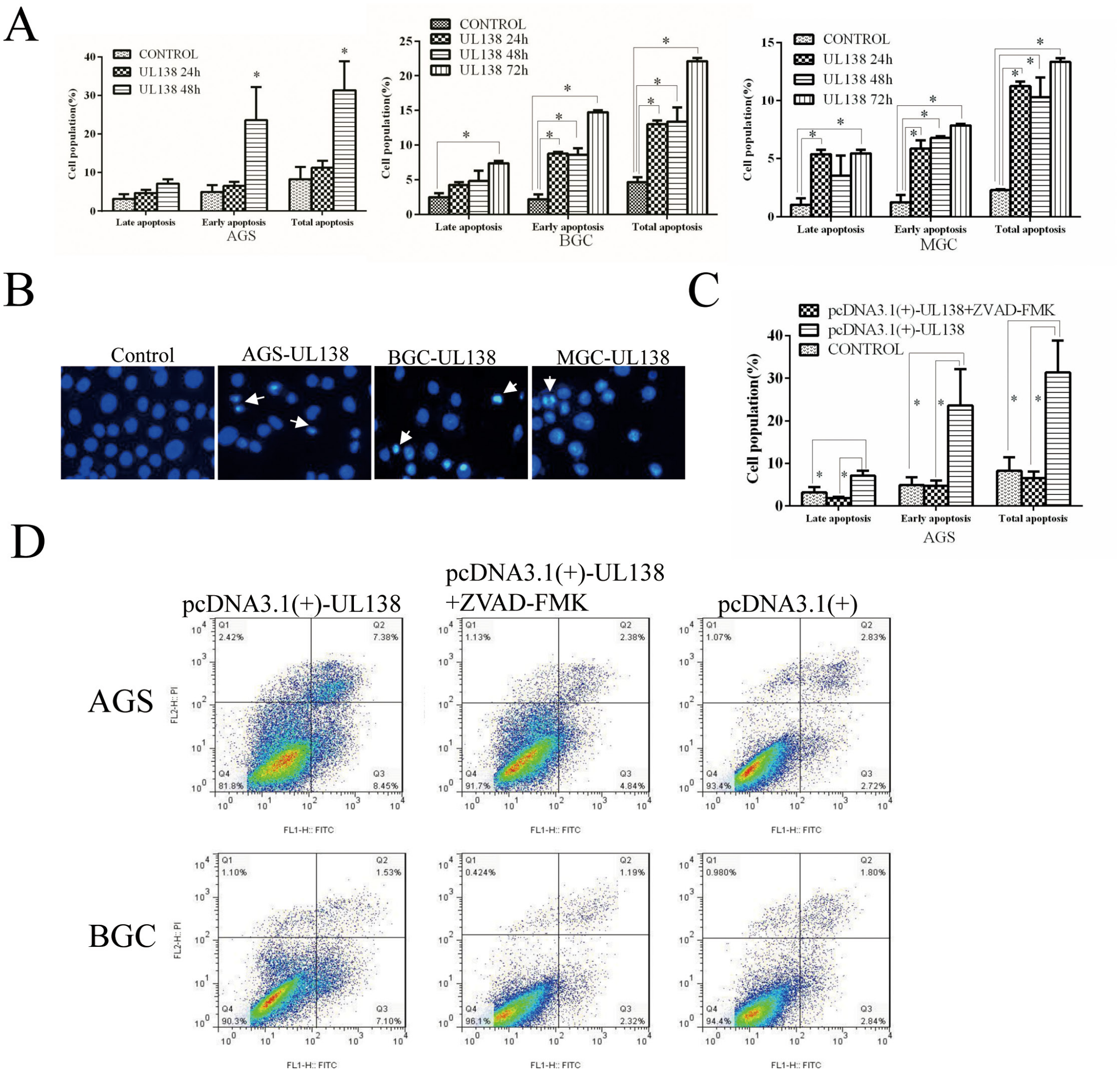

Supplementary Figure S5: UL138-caused apoptosis of gastric cancer cells was reversed by z-VAD-FMK. (**A**) GC cells were transfected with pcDNA3.1(+)-UL138 (UL138) or pcDNA3.1(+) (control) and were prepared for apoptosis assay by flow cytometry at indicated times post transfection. Data was presented as means ± SEM of three independent experiments. Statistical analyses of the number of apoptotic cells were shown accordingly. (**B**) GC cells were stained with Hoechst 33258 and observed under a fluorescence microscope (Magnification, ×200) at 48 hr post UL138 transfection. Arrows indicated nuclear fragmentation in apoptotic cells. (**C, D**) AGS and BGC-823 cells were transfected with pcDNA3.1(+)-UL138 + ZVAD-FMK or pcDNA3.1(+)-UL138 or pcDNA3.1(+) (control) and were prepared for apoptosis assay by flow cytometry at 48 hr post transfection. Data of AGS was presented as means ± SEM of three independent experiments. Statistical analyses of the number of apoptotic cells were shown accordingly.

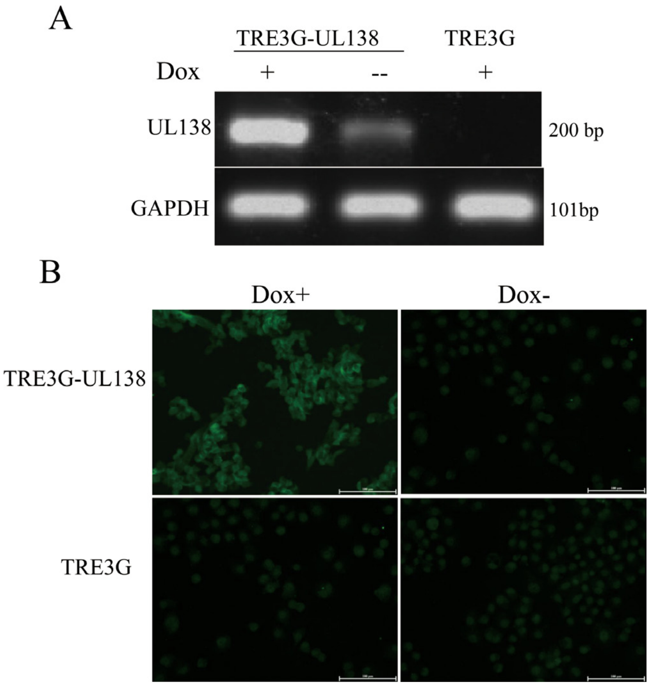

Supplementary Figure S6: Establishment of stably transfected BGC-823 cell with a controllable gene expression of UL138. TRE3G-UL138 referred to BGC-823 cells containing recombinational plasmids pTRE3G-UL138 and pCMV-Tet3G. TRE3G referred to BGC-823 cells containing two plasmids pTRE3G and pCMV-Tet3G and was set as control. (**A**) PCR detection of the expression of UL138 mRNA in constructed BGC-UL138 cells after dox induced for 48 hr. (**B**) Detection of pUL138 protein in constructed BGC-UL138 cells at 48 hr post dox induction by fluorescence microscopy (100× magnification).

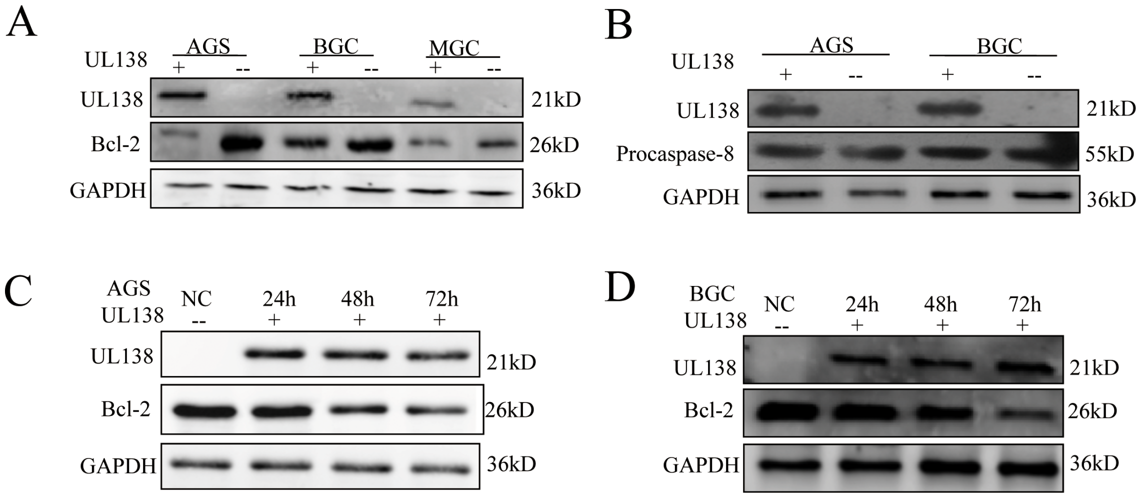

Supplementary Figure S7: Expression of apoptosis-related proteins in gastric cancer cells expressing UL138. (**A**) GC cells were transfected with pcDNA3.1(+)-UL138 or pcDNA3.1(+) (indicated as UL138+/−). Expressional changes of Bcl-2 in gastric cancer cells expressing UL138 were determined by Western blot at 48 hr post transfection. GAPDH served as a loading control. (**B**) There was no expressional change of Procaspase-8 in gastric cancer cells expressing UL138 were determined by Western blotting at 48 hr post transfection. (**C, D**) Expression changes of UL138 and Bcl-2 at indicated times in AGS and BGC-823 gastric cancer cells by Western blot.

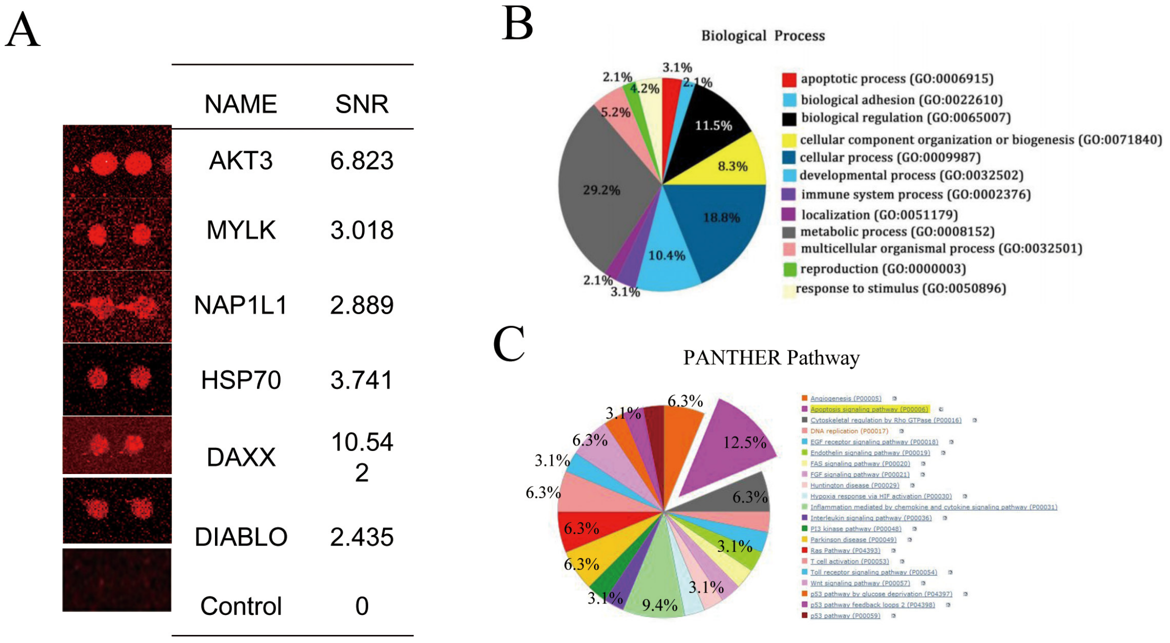

Supplementary Figure S8: pUL138-interacting protein representatives were generated by a human proteome microarray (A) and analyzed by PANTHER classification for both biological processes (B) and signaling pathways (C).

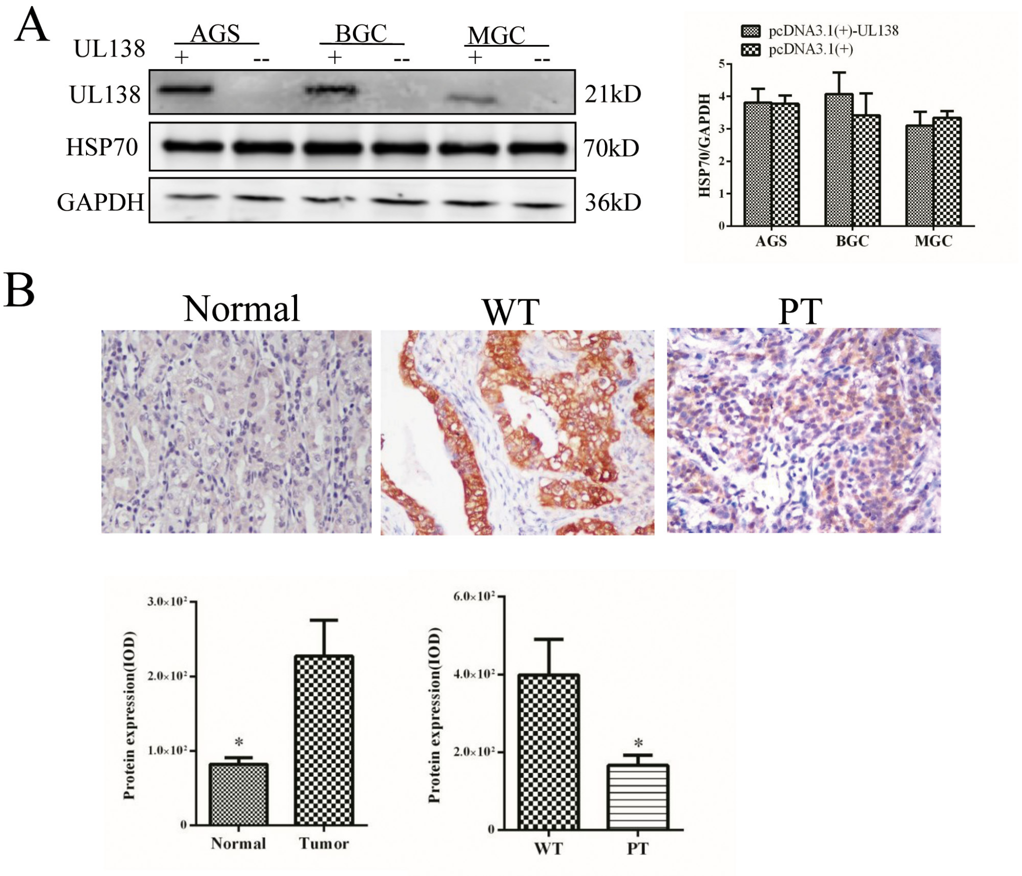

Supplementary Figure S9: Expression of HSP70 in gastric cancer cells and tissues. (**A**) GC cells were transfected with pcDNA3.1(+)-UL138 or pcDNA3.1(+) (indicated as UL138+/−). Expressional changes of HSP70 in GC cells were detected at 48 hr post transfection by Western blot and followed by quantitated densitometric analysis using ImageJ software. GAPDH served as a loading control. (**B**) The HSP70 expression in gastric cancer and adjacent normal tissues were detected by semi-quantitative immunohistochemistry. WT, well differentiated tumors; PT, poorly or none differentiated tumors. Statistical analysis of immunohistochemical signals were shown as column. $n = 20$. $^*P < 0.05$.

Supplementary Table S1: Annotation of UL138 binding genes by GO and KEGG terms

Category	Term	Number of genes	Fold Enrichment	*p* value*
GO	GO:0043067~regulation of programmed cell death	40	2.389	6.03E-07
	GO:0010941~regulation of cell death	40	2.380	6.65E-07
	GO:0042981~regulation of apoptosis	39	2.352	1.28E-06
	GO:0043066~negative regulation of apoptosis	23	3.150	4.04E-06
	GO:0051726~regulation of cell cycle	22	3.223	4.91E-06
	GO:0043069~negative regulation of programmed cell death	23	3.106	5.05E-06
KEGG	hsa04640:Hematopoietic cell lineage	11	4.747	8.43E-05
	hsa05200:Pathways in cancer	17	1.924	1.35E-02
	hsa04060:Cytokine-cytokine receptor interaction	14	1.983	2.22E-02
	hsa05222:Small cell lung cancer	7	3.093	2.42E-02
	hsa04621:NOD-like receptor signaling pathway	6	3.592	2.43E-02
	hsa05219:Bladder cancer	5	4.419	2.50E-02
	hsa04115:p53 signaling pathway	6	3.275	3.45E-02
	hsa04110:Cell cycle	8	2.375	4.92E-02

*:Statistical significance of the difference between the fraction of UL138 interacting genes assigned to this GO term and the fraction of all genes within the homo sapiens set assigned to this GO term.

Supplementary Table S2: Sixty eight UL-138-interacting protein candidates

Number	Protein Name	Gene Bank
1	QDPR	BC000576.2
2	C5orf3	BC011414.1
3	KIAA0515	BC012289.1
4	DAPP1	BC012924.1
5	ZMYM3	BC013009.2
6	SEPT9	BC021192.2
7	DKFZP686A01247	BC023546.2
8	XRCC1	BC023593.2
9	NEDD4L	BC032597.1
10	HSPA1L	BC034483.1
11	Zfp185	BC039772

12	HS1BP3	BC050636.1
13	MARCKSL1	BC066915.1
14	SRA1	BC067895.1
15	PAK2	BC069613.1
16	FLJ39378	BC089444.1
17	IDH1	BC093020.1
18	SLC4A1AP	BC098302.1
19	MOGAT2	BC103878.1
20	SMARCC1	BC113465
21	MYLK	ENST00000361290
22	LIG1	NM_000234.1
23	SUHW4	NM_001002844.1
24	PLCXD3	NM_001005473.2
25	TCOF1	NM_001008657.1
26	KIAA1189	NM_001009959.1
27	TCEAL5	NM_001012979.1
28	TIRAP	NM_001039661.1
29	GAGE1	NM_001040663.1
30	PPP1R12B	NM_001167858.1
31	CDKN2C	NM_001262.2
32	DAXX	NM_001350.3
33	MYL5	NM_002477.1
34	PLEK	NM_002664.1
35	SMARCC2	NM_003075.2
36	TPD52L2	NM_003288.2
37	NAP1L1	NM_004537.3
38	RBM39	NM_004902.2
39	CHES1	NM_005197.2
40	HCLS1	NM_005335.3
41	AKR1D1	NM_005989.2
42	VCP	NM_007126.2
43	R3HDM2	NM_014925.2
44	CYB5R4	NM_016230.3
45	HAO1	NM_017545.2
46	TERF2IP	NM_018975.2

47	PCNP	NM_020357.1
48	DLG3	NM_021120.2
49	EIF4H	NM_022170.1
50	EFCBP1	NM_022351.2
51	SMYD3	NM_022743.1
52	C9orf58	NM_031426.2
53	RBM17	NM_032905.3
54	ATCAY	NM_033064.3
55	SNX21	NM_033421.2
56	SRXN1	NM_080725.1
57	XAGE3	NM_130776.1
58	DIABLO	NM_138930.2
59	PSMA1	NM_148976.1
60	CAST	NM_173060.2
61	RABL3	NM_173825.2
62	FAM9C	NM_174901.3
63	C12orf61	NM_175895.2
64	AKT3	NM_181690.1
65	ARS2	NM_182800.2
66	C6orf141	Q5SZD1
67	LOC729447	XM_001130991.1
68	LOC339804	XM_291016.3

Supplementary Table S3: GO terms enriched in the UL138 interactome

Term	Term	Description	Count[a]	%[b]	*p* value[c]
Biological process	GO:0006259	DNA metabolic process	7	1.160862355	0.01150115
	GO:0006974	response to DNA damage stimulus	6	0.995024876	0.012895062
	GO:0033554	cellular response to stress	7	1.160862355	0.019055165
	GO:0046487	glyoxylate metabolic process	2	0.331674959	0.022411734
	GO:0006260	DNA replication	4	0.663349917	0.034712341
	GO:0051276	chromosome organization	6	0.995024876	0.035251512
Cellular component	GO:0031981	nuclear lumen	13	2.155887231	2.98E-04
	GO:0005654	nucleoplasm	10	1.658374793	4.69E-04
	GO:0070013	intracellular organelle lumen	13	2.155887231	0.001906619
	GO:0043233	organelle lumen	13	2.155887231	0.002324187
	GO:0031974	membrane-enclosed lumen	13	2.155887231	0.002751218
Molecular function	GO:0000166	nucleotide binding	16	2.653399668	0.012835413
	GO:0008289	lipid binding	6	0.995024876	0.024650768
	GO:0005543	phospholipid binding	4	0.663349917	0.027527085

[a]Number of UL138 interacting proteins.

[b]Percentage of mapped proteins associated with each term.

[c]Statistical significance of the difference between the fraction of UL138 interacting proteins assigned to this GO term and the fraction of all proteins within the human protein set assigned to this GO term.

The top enriched GO biological process and pathway analysis enriched in the UL138 interactome are listed.

Supplementary Table S4: Animal groups and administration

Animal Group	Administration	Dox*	*n*
TRE UL138+	pTRE3G-UL138&pCMV-Tet 3G	+	7
TRE UL138−	pTRE3G-UL138&pCMV-Tet 3G	−	7
TRE +	pTRE3G&pCMV-Tet 3G	+	7

*Dox+, 1 mg/ml in drinking water; Dox−, without Dox in drinking water.
